# Dyshidrosiform Bullous Pemphigoid: A Palmar Variant of a Common Disease

**DOI:** 10.7759/cureus.75639

**Published:** 2024-12-13

**Authors:** Alexa Lum, Mitchell Brady

**Affiliations:** 1 Dermatology, Michigan State University College of Osteopathic Medicine, East Lansing, USA; 2 Dermatology, Corewell Health Farmington Hills Hospital, Farmington Hills, USA

**Keywords:** autoimmune blistering diseases, bullous pemphigoid (bp), dyshidrosiform bullous pemphigoid, dyshidrotic, subepidermal blister

## Abstract

Dyshidrosiform bullous pemphigoid (DBP) is a rare variant of bullous pemphigoid (BP) that mainly affects elderly patients and presents with tense bullae formation on the palms, soles, or both palms and soles. This case report describes an 87-year-old woman who was evaluated in the hospital for a month-long erythematous and pruritic rash on most of her body that eventually manifested into tense blisters on the palms. DBP can pose a challenge to clinicians as it can resemble a variety of different vesicular diseases. Therefore, clinical suspicion, as well as confirmatory testing with direct immunofluorescence (DIF), can aid clinicians in making a diagnosis of DBP. The mainstay of treatment is oral corticosteroids plus an immunosuppressant, but some monoclonal antibodies have shown promising efficacy in the treatment of more refractory cases of DBP. Additional research that focuses on the role of these monoclonal antibodies in the treatment of DBP is needed to determine their therapeutic benefit.

## Introduction

Bullous pemphigoid (BP) is a common autoimmune bullous disorder that involves antibodies that target the hemidesmosome complex of the skin and, subsequently, cause acantholysis and subepidermal blistering [1]. BP is more prevalent in the elderly, with females typically being more affected than males and with an increase in frequency after age 70 [2]. BP has also been seen to increase in frequency in patients with neurodegenerative disorders, such as dementia, Parkinson’s disease, and strokes, as well as with the use of certain medications, such as sulfa-containing medications, angiotensin-converting enzyme inhibitors (ACEi), and non-steroidal anti-inflammatory drugs (NSAIDs) [1]. Diagnosis of BP in the early stages can be difficult, as it typically presents with non-specific urticaria or eczematous plaques that are very pruritic. The later and more classic presentation of BP consists of pruritic and tense blisters that favor the trunk and flexural areas of the extremities and spare the mucosal surfaces [1]. However, there is a rare variant of BP that presents with tense, pruritic bullae that favor the palms and soles, appropriately named dyshidrosiform bullous pemphigoid (DBP). Since the first record of DBP in 1979 by Doctors Levine, Freilich, and Barland [3], there have been less than 100 cases of DBP reported in the literature. In this report, we present a case of a woman who developed subepidermal blisters on her palms after a month-long history of a pruritic rash that covered a significant percentage of her body and did not fully respond to oral corticosteroid treatment. This case highlights the necessity of differentiating DBP from other vesicular diseases and provides guidance on alternative therapy options when a patient fails to respond to the standard treatment.

## Case presentation

**Figure 1:**
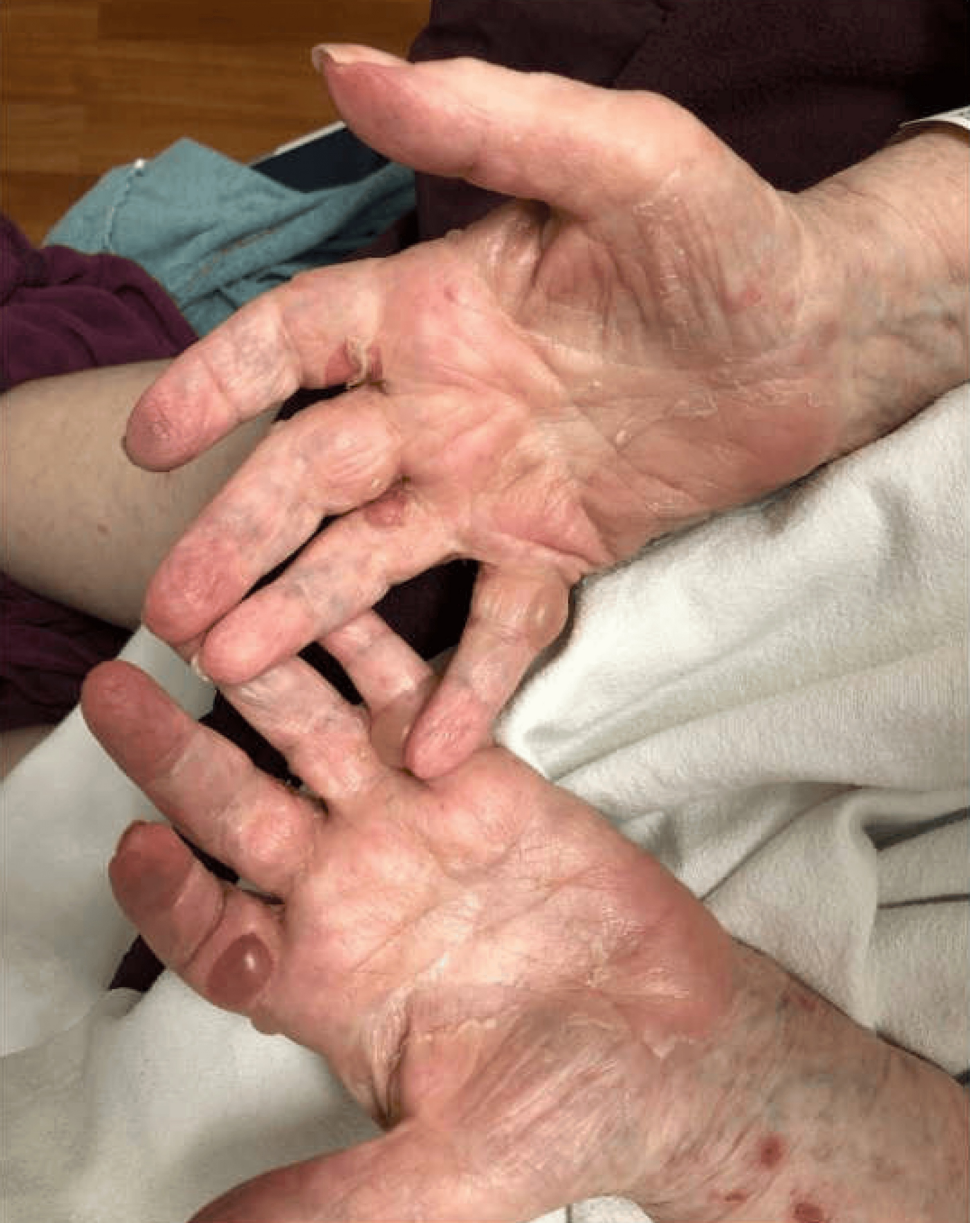
Multiple, tense 1-cm bullae and erosions on the palmar aspect of the fingers

An 87-year-old woman with a past medical history of type 2 diabetes mellitus, paroxysmal atrial fibrillation, chronic obstructive pulmonary disease (COPD), coronary artery disease, depression, and sick sinus syndrome with pacemaker implantation presented to the emergency department with a worsening rash on both of her wrists, hands, back, lower abdomen, and thighs of one-month duration. A month prior to her presentation, she developed a pruritic rash on both of her wrists, hands, back, lower abdomen, and thighs, which prompted her to seek treatment at urgent care. She was prescribed a five-day course of oral steroids and was advised to follow up with her dermatologist. Dermatology initially performed a shave biopsy on the left wrist and left mid-upper back, which showed lichenoid interface dermatitis with eosinophils consistent with a lichenoid hypersensitivity reaction and a bullous hypersensitivity reaction, respectively. Due to the inconclusive biopsy results, she was advised to return in a week to the dermatology office for another biopsy with direct immunofluorescence (DIF). The DIF from a punch biopsy of her left lower back showed linear deposition of immunoglobulin G (IgG) and C3 along the basement membrane zone, consistent with a diagnosis of BP. She was then initiated on minocycline 100 mg twice daily, nicotinamide 500 mg twice, and betamethasone cream twice daily for new blisters. Over the course of the month, the rash and pruritus showed minimal improvement. She then experienced a flare with a new blister formation on her hands, which prompted her visit to the emergency department. Physical exam was notable for multiple tense bullae and erosions measuring 1 cm on the palmar aspect of the fingers on both hands (Figure 1), as well as erythematous patches and plaques on the back, thighs, and lower abdomen. There was no mucosal involvement noted. Labs were remarkable for a slightly elevated white blood cell count with eosinophils (Table 1). She was subsequently treated with a daily high-dose oral corticosteroid during her hospital stay and discharged with a steroid taper over the course of three weeks, as well as the continuation of her prior medications (minocycline, nicotinamide, and topical betamethasone cream). Unfortunately, the patient was unable to follow up, and there was no confirmed resolution of the bullae from her dermatology office.

**Table 1:** Laboratory investigations WBC, white blood cells; Abs Eos Manual, absolute eosinophils manual

Test	Observed value	Reference range
WBC	12.2 bil/L	3.3-10.7 bil/L
Abs Eos Manual	3.4 bil/L	0.0-0.5 bil/L

## Discussion

DBP is a rare subtype of BP that presents as pruritic and occasionally hemorrhagic blisters on the palms, soles, or both palms and soles of elderly patients with an established diagnosis of BP [4]. The palmar and plantar bullae can form as a result of bullae formation on other sites of the body or can form independently [5]. Similar to the pathogenesis of BP, DBP occurs because of circulating and tissue-bound autoantibodies targeted at BP antigen 230, BP antigen 180, or both simultaneously [4]. BP antigen 230 and BP antigen 180 are both components of the hemidesmosome complex, which plays a pivotal role in binding the cytoskeleton of basal keratinocytes to the papillary dermis [6]. Disruption of this complex leads to the classic subepidermal blisters that are seen with BP and DBP.

Doctors Levine, Freilich, and Barland were the first to report on the findings of DBP in 1979 when they examined an elderly male with a vesicular eruption on the hands and tense bullae on the feet [3]. Since then, there have been less than 100 cases of the rare variant reported in the literature. The morphology of DBP can pose a challenge to diagnosis due to its close resemblance to multiple other vesicular diseases, such as dyshidrotic eczema, allergic and irritant contact dermatitis, epidermolysis bullosa acquisita (EBA), cutaneous T-cell lymphoma (vesicular palmoplantar variant), bullous dermatophyte infection, and bullous lichen planus (BLP) [5]. However, DBP is distinguished from other vesicular diseases by DIF, which shows linear deposition of complement component 3 (C3) and IgG along the dermoepidermal junction [5]. Further serration analysis can differentiate DBP from EBA, as DBP does not reveal the classic “u-serrated” pattern that is exclusively seen with EBA [7]. In contrast to DBP and EBA, dyshidrotic eczema shows spongiosis on histopathology and is usually diagnosed based on clinical suspicion [8]. The histopathology of BLP typically shows vacuolar degeneration of the basal layer, also known as Max-Joseph spaces, which subsequently causes the formation of tense bullae that are intrabasal in nature [9].

The standard treatment for DBP is immunosuppression with agents such as topical and systemic corticosteroids, tetracycline antibiotics, dapsone, azathioprine, and mycophenolate mofetil [5]. Joly et al. found that out of 188 patients who had extensive BP, topical corticosteroids were a superior treatment compared to oral corticosteroids and should be considered as the first-line treatment [10]. However, most cases of DBP require management with a systemic corticosteroid agent with a starting dose of 0.5-0.75 mg/kg/day that is continued until there is resolution of the existing lesions and no new blister formation [11]. Once clearance is achieved, the systemic corticosteroid can be gradually tapered to 5 mg daily for five to six months [11]. Dupilumab, a recombinant human IgG4 monoclonal antibody that blocks the interleukin-4 receptor alpha (IL-4Ra), is a treatment option considered in DBP patients who do not respond to the standard treatments listed above. It is theorized that dupilumab treats refractory DBP by inhibiting interleukin-4 (IL-4) and interleukin-13 (IL-13) signal transduction, therefore reducing T helper type 2 (Th2)-related cytokines such as IL-4, IL-13, and IL-31, which are thought to be increased in both BP and DBP [12]. The increase in Th2 cytokines also plays a significant role in eosinophilia, as seen in our patient. Other promising treatments for DBP include rituximab, an anti-CD20 monoclonal antibody, and omalizumab, a monoclonal antibody targeting immunoglobulin E (IgE) [13-14]. However, further research, specifically randomized clinical trials, is needed to determine the therapeutic benefit of dupilumab, rituximab, and omalizumab on DBP.

## Conclusions

In conclusion, DBP remains a challenge to diagnose due to its close resemblance to other vesicular and dyshidrotic dermatoses. However, with an accurate timeline of the development of the bullae, diagnostic tools such as DIF, and clinical suspicion, DBP can be more efficiently and effectively diagnosed, ensuring that the correct treatment will be given to the patient. The use of monoclonal antibodies, such as dupilumab, rituximab, and omalizumab, may be a promising treatment option for more diffuse or refractory cases of DBP due to their ability to specifically target Th2 cytokines that are implicated in the disease process and to potentially decrease the length of time on corticosteroids.
